# Asthma with recurrent middle lobe syndrome in children: Clinical features and lung function patterns

**DOI:** 10.3389/fped.2023.1113652

**Published:** 2023-02-06

**Authors:** Yong Feng, Haoting Yu, Xin Liu, Ning Chen, Yunxiao Shang, Han Zhang

**Affiliations:** Department of Pediatrics, Shengjing Hospital of China Medical University, Shenyang, China

**Keywords:** asthma, children, atelectasis, middle lobe syndrome, lung function

## Abstract

**Background:**

Middle lobe syndrome (MLS) is a complication of childhood asthma. This study aimed to compare the clinical features and lung function between asthmatic children with recurrent MLS and transient right middle lobe (RML) and/or lingula atelectasis.

**Methods:**

This study retrospectively analyzed asthmatic children with RML and/or lingula atelectasis between 2010 and 2020 using data from the pediatric pulmonary department. According to the episodes of atelectasis, children were divided into recurrent (≥2 episodes) and non-recurrent (only 1 episode) MLS groups, to compare clinical features and lung function. Spirometry during acute asthma exacerbation and stable stages were recorded, and variations were calculated.

**Results:**

A total of 35 children with asthma and RML and/or lingula atelectasis were included, 15 of whom had recurrent MLS. The recurrent MLS group had a higher proportion of girls, infections, family allergy history, severe asthma, severe exacerbation, and higher levels of total IgE than the non-recurrent MLS group (*P *< 0.05). The recurrent MLS group had a significantly higher % predicted and z-scores for forced expiratory volume in 1 s (FEV_1_) and forced vital capacity (FVC), a greater proportion of high FEV_1_ and higher variations in FEV_1_ and FVC than that in the non-recurrent group (*P* < 0.05). After excluding children with mild to moderate asthma in the recurrent MLS group, the differences in clinical features disappeared, but the results regarding lung function remained similar, when compared to severe asthma patients without RML and/or lingula atelectasis.

**Conclusions:**

Childhood asthma with recurrent MLS has more frequent severe asthma and exacerbation but high lung function and variations.

## Introduction

Atelectasis is an incomplete expansion of the lung parenchyma, which is proven radiographically. Atelectasis is a common complication of pulmonary and extrapulmonary diseases affecting children of all ages, especially younger children (1–3). The right middle lobe (RML) and lingula are susceptible to atelectasis due to poor collateral ventilation caused by their anatomical features, including the relatively narrow and long RML bronchus, sharp take-off angle, deep fissures with scanty parenchymal bridges, and poor development of the pores of Kohn and canals of Lambert (4). Most atelectasis can resolve spontaneously or with treatment, but in some cases, atelectasis persists or recurs, especially in the RML or lingula, which is termed middle lobe syndrome (MLS) (2).

MLS is a relatively uncommon and under-recognized clinical entity that was first described by Graham et al. in 1948 (5). Asthma is a common cause of RML atelectasis and MLS (2, 6). Sekerel et al. reported 3,528 asthmatic children, of which 56 (1.62%) developed MLS (6). It is not yet fully understood why asthma is prone to MLS. Children with asthma and MLS are more often younger girls and exhibit non-atopic asthma (1, 6). Poor asthma control might contribute to the occurrence of MLS. Soyer et al. revealed that asthmatic children with RML atelectasis persistent for more than two weeks, had lower Childhood Asthma Control Test scores and less use of anti-inflammatory medications (2). Sekerel et al. found that asthmatic children with MLS took a significantly longer time to recover from symptoms than asthma controls, suggesting uncontrolled asthma (6). The relationship between severity of asthma and the occurrence of MLS still needs to be evaluated.

MLS can be divided into recurrent and persistent types according to the resolution and episode of atelectasis (4, 7). Bacterial infections have been suggested as a factor associated with persistent MLS in asthmatic children (8), which was not detected in asthmatic children with MLS in later studies (1, 6). Thus, the recurrent and persistent types of MLS may have different pathophysiological and clinical features. Few studies had evaluated the features of the recurrent MLS in children with asthma. Recently, Comberiati et al. reported an interesting phenomenon in children with severe asthma, who had a forced expiratory volume in 1 s (FEV_1_) > 100% predicted during the clinically stable stage but can experience catastrophic reductions in airflow and life-threatening asthma exacerbation (9). We speculated that this phenomenon might also present in the recurrent MLS in asthmatic children. Therefore, we sought to determine the clinical and lung function characteristics of childhood asthma with recurrent MLS.

## Methods

### Study subjects

A retrospective analysis was conducted using electronic medical records from the pediatric pulmonary department of the Shengjing Hospital of China Medical University between January 2010 and January 2020. Children who were aged < 14 years and hospitalized with asthma and radiographically proven RML and/or lingula lobe atelectasis, were included in this study. Children were further evaluated and divided into the recurrent and non-recurrent MLS groups according to the resolution and episode of atelectasis (4, 7). Recurrent MLS was defined as the presence of at least two episodes of radiographically confirmed RML and/or lingula atelectasis with radiographic clearance between the episodes. Children with only one episode of RML and/or lingula atelectasis were classified as non-recurrent MLS group. All children included should have at least one radiographic follow-up during 1–12 months after the first atelectasis, to evaluate the radiographic clearance. Both chest x-rays and chest computed tomography scans were reviewed. Asthma was diagnosed by a physician, based on a history of respiratory symptoms, such as recurrent wheezing, cough, breathlessness or chest tightness, and reversible airflow limitation, indicated by favorable responses to inhaled corticosteroids (ICS) and/or inhaled bronchodilators or improvement of at least 12% in FEV_1_ following bronchodilator administration (10). Severe asthma was defined as asthma that is uncontrolled despite high dose ICS plus a second controller, or that requires this therapy to remain controlled (10, 11). Children with severe asthma but without RML and/or lingula atelectasis were also included and classified as severe asthma control group. This study was approved by the Ethics Committee of the Shengjing Hospital of China Medical University (No. 2022PS679K). The need for informed consent was waived off as data were anonymized.

### Study methods

Demographic data, clinical characteristics, laboratory findings, and bronchoscopy findings were collected retrospectively. Clinical characteristics included personal and family history of atopy, age at asthma diagnosis, asthma duration, treatment duration, pediatric intensive care unit (PICU) admission, duration of complaints, symptoms, asthma severity, and exacerbation severity. Laboratory findings included white blood cell and eosinophil counts, serum vitamin D levels, respiratory pathogen diagnosis, serum total immunoglobulin E (IgE) levels, and serum specific allergen testing. Mycoplasma pneumoniae (MP) infection was diagnosed based on positive MP-immunoglobulin M test of serum and positive polymerase chain reaction (PCR) test of naso/oropharyngeal swabs, sputum or bronchoalveolar lavage (BAL) fluid. Bacterial infection was diagnosed based on positive culture of blood or BAL fluid. Virial infection was diagnosed based on positive PCR test of naso/oropharyngeal swabs, sputum or BAL fluid, for common respiratory viruses, including adenovirus, respiratory syncytial virus, influenza A, influenza B, and parainfluenza viruses 1–3. Fractional exhaled nitric oxide (FeNO) was measured using an online single-breath method (12, 13).

Bronchoscopy findings were collected, including macroscopic evaluation of the tracheobronchial anatomy, mucus secretion and inflammation, and differential cell counts in BAL fluid. Mucosal inflammation was defined as mucosal edema, hyperemia, and/or longitudinal mucosal folds. BAL fluid from the affected lobe was analyzed for differential cell counts (macrophages, lymphocytes, neutrophils, eosinophils, and epithelial cells) and microbiology. Presence of more than 10% neutrophils (14, 15), 15% lymphocytes (16), and 1% eosinophils (17) were defined as significant neutrophilic, lymphocytic, and eosinophilic inflammation, respectively.

### Spirometry measurement

Spirometry was performed using a pneumotachograph-type spirometer (MasterScreen Pneumo, Jaeger, Hoechberg, Germany) according to recommendations adapted for children (18, 19). For quality control, all recruited tests were reviewed and selected by two investigators according to recommendations (18, 19). Data were converted using the 2012 Global Lung Function Initiative reference equations for Northeast Asians (20) and expressed as % predicted and *z*-scores. Both pre- and post-bronchodilator (BD) spirometry data were collected, including FEV_1_, forced vital capacity (FVC), FEV_1_ to FVC ratio (FEV_1_/FVC), mean forced expiratory flow between 25% and 75% of FVC (FEF_25–75_), and instantaneous forced expiratory flow at 75% of FVC (FEF_75_). Spirometry performed during acute asthma exacerbation and stable stages were recruited separately. Spirometry during acute asthma exacerbation should be performed on or shortly after admission and before bronchoscopy. Variations in FEV_1_ and FVC between acute and stable stages, were calculated as follows: (stable−acute)/mean (stable + acute) × 100.

### Statistical analysis

Descriptive analysis was conducted to understand the clinical and lung function characteristics of children with recurrent MLS and asthma. Data distributions were assessed using the Shapiro–Wilk test. Normally distributed continuous variables were expressed as mean ± standard deviation and non-parametric continuous variables as medians (interquartile ranges). Categorical variables were expressed as frequencies with percentages. To compare differences between groups, the Student's t-test and Mann–Whitney U-test were used for parametric and non-parametric continuous data, respectively. The chi-square test or Fisher's exact test was used to determine the significance of differences in categorical data between the groups. Two-tailed *p*-values of < 0.05 were considered statistically significant. Statistical analysis was performed using IBM SPSS Statistics version 20 (IBM, Armonk, NY, United States) and GraphPad Prism version 8 (GraphPad Software Inc., San Diego, CA, United States).

## Results

### Clinical features of asthmatic children with recurrent MLS

Thirty-five asthmatic children with RML and/or lingula atelectasis were included, of whom 15 had recurrent MLS. The clinical features of the children with recurrent MLS and non-recurrent MLS are summarized in Table 1. In the recurrent MLS group, the average interval of two episodes was 8.0 ± 3.7 months (median 7.5 months, range 2.0–14.2 months). The recurrent MLS group had more girls than the non-recurrent MLS group (73.3% vs. 35.0%, *P* = 0.025). The recurrent MLS group had longer duration of complaints, higher proportions of infection and family allergy history, and higher total IgE levels than the non-recurrent MLS group; however, no significant differences were found in personal atopy, eosinophil count, sensitization to allergens, and FeNO values between the two groups. Regarding the characteristics of asthma, compared to children in the non-recurrent MLS group, children with recurrent MLS had a significantly greater proportion of severe asthma and severe exacerbation, but similar age at asthma diagnosis and similar duration of asthma and treatment.

**Table 1:** Clinical features of asthmatic children with RML and/or lingula atelectasis stratified by recurrent MLS.

Characteristics	Recurrent MLS (*n* = 15)	Non-recurrent MLS (*n* = 20)	*P*-value
Female sex	11 (73.3%)	7 (35.0%)	0.025
Age at asthma diagnosis (years)	4.4 (3.8–5.5)	4.5 (3.6–6.1)	0.944
Asthma duration (years)	1.5 (0.9–3.0)	1.1 (0.2–2.1)	0.359
Asthma treatment duration (years)	0.9 (0.0–2.8)	0.6 (0.0–1.0)	0.381
Duration of complaints (days)	3.5 (3.0–5.5)	10.0 (6.0–17.5)	0.003
**Symptoms**			
Fever	5 (33.3%)	10 (50.0%)	0.324
Cough	14 (93.3%)	17 (85.0%)	0.619*
Wheezing	13 (86.7%)	14 (70.0%)	0.419*
Dyspnea	9 (60.0%)	4 (20.0%)	0.015
Postevent follow-up (years)	3.2 (1.7–5.6)	2.9 (1.5–5.6)	0.714
Severe asthma	11 (73.3%)	1 (5.0%)	<0.001
Severe exacerbation	9 (60.0%)	4 (20.0%)	0.015
PICU admission	5 (33.3%)	1 (5.0%)	0.064*
Personal allergy history	11 (73.3%)	16 (80.0%)	0.700*
Family allergy history	12 (80.0%)	6 (30.0%)	0.003
White blood cell count (10^9^/l)	10.0 ± 4.5	10.6 ± 3.9	0.667
Eosinophil count (10^9^/l)	0.2 (0.0–0.4)	0.1 (0.1–0.4)	0.706
Vitamin D (ng/mL)	19.0 (14.5–25.0)	23.3 (13.2–36.2)	0.429
Infection	13 (86.7%)	10 (50.0%)	0.024
MP	10 (66.7%)	8 (40.0%)	0.118
Bacteria	3 (20.0%)	0 (0.0%)	0.070*
Virus	0 (0.0%)	2 (10.0%)	0.496*
Sensitization to ≥1 allergen	9 (60.0%)	9 (45.0%)	0.380
Mites	3 (20.0%)	6 (30.0%)	0.700*
Foods	8 (53.3%)	5 (25.0%)	0.086
Molds	4 (26.7%)	2 (10.0%)	0.367*
Pets	1 (6.7%)	2 (10.0%)	1.000*
Plants	2 (13.3%)	3 (15.0%)	1.000*
Total IgE level (IU/ml)	234.1 (62.3–490.5)	62.6 (31.3–184.0)	0.022
FeNO (ppb)	14.6 ± 9.6 (*n* = 12)	12.7 ± 6.7 (*n* = 12)	0.606

Data are presented as *n* (%), mean ± standard deviation or median (interquartile range). RML, right middle lobe; MLS, middle lobe syndrome; PICU, pediatric intensive care unit; MP, Mycoplasma pneumoniae; IgE, immunoglobulin E; FeNO, fractional exhaled nitric oxide.

* Comparisons were made by Fisher's exact test.

In addition, to evaluate the influence of severe asthma on the above findings, we further performed an analysis comparing 11 subjects with severe asthma and recurrent MLS, with 10 severe asthma controls (Table 2). No significant differences were found between the two groups in terms of clinical features, except for the presence of slightly higher proportion of girls (*P* = 0.086) and a shorter asthma treatment duration (*P* = 0.072) in children with recurrent MLS than in severe asthma controls.

**Table 2:** Clinical features of children with severe asthma stratified by recurrent MLS.

Characteristics	Severe asthma with recurrent MLS (*n* = 11)	Severe asthma control (*n* = 10)	*P*-value
Female sex	8 (72.7%)	3 (30.0%)	0.086*
Age at asthma diagnosis (years)	4.1 (2.1–6.3)	4.4 (3.8–5.5)	0.888
Asthma duration (years)	1.5 (0.9–5.2)	3.8 (3.0–7.1)	0.072
Asthma treatment duration (years)	0.9 (0.5–2.9)	2.0 (0.8–3.3)	0.395
Duration of complaints (days)	3.0 (3.0–5.0)	4.5 (2.8–7.8)	0.519
**Symptoms**			
Fever	2 (18.2%)	2 (20.0%)	1.000*
Cough	10 (90.9%)	10 (100.0%)	1.000*
Wheezing	10 (90.9%)	8 (80.0%)	0.419*
Dyspnea	6 (64.5%)	7 (70.0%)	0.659*
Severe exacerbation	6 (64.5%)	7 (70.0%)	0.659*
PICU admission	4 (36.4%)	1 (10.0%)	0.311*
Personal allergy history	8 (80.0%)	8 (72.7%)	1.000*
Family allergy history	10 (90.9%)	9 (90.0%)	1.000*
White blood cell count (10^9^/l)	9.1 ± 3.7	8.2 ± 1.6	0.512
Eosinophil count (10^9^/l)	0.2 (0.0–0.4)	0.4 (0.2–0.9)	0.169
Vitamin D (ng/ml)	19.8 (14.2–25.2)	19.8 (16.2–29.6)	0.725
Infection	9 (81.8%)	4 (40.0%)	0.080*
MP	7 (63.3%)	3 (30.0%)	0.198*
Bacteria	2 (18.2%)	0 (0.0%)	0.476*
Virus	0 (0.0%)	1 (10.0%)	0.476*
Sensitization to ≥1 allergen	6 (54.5%)	7 (70.0%)	0.659*
Mites	1 (9.1%)	3 (30.0%)	0.311*
Foods	5 (45.5%)	4 (40.0%)	1.000*
Molds	2 (18.2%)	3 (30.0%)	0.635*
Pets	0 (0.0%)	2 (20.0%)	0.214*
Plants	1 (9.1%)	3 (30.0%)	0.311*
Total IgE level (IU/ml)	181.5 (62.0–264.6)	141.9 (97.1–539.6)	1.000
FeNO (ppb)	14.6 ± 9.6 (*n* = 9)	12.7 ± 6.7 (*n* = 10)	0.606

Data are presented as *n* (%), mean ± standard deviation or median (interquartile range). MLS, middle lobe syndrome; PICU, pediatric intensive care unit; MP, *Mycoplasma pneumoniae*; IgE, immunoglobulin E; FeNO, fractional exhaled nitric oxide.

* Comparisons were made by Fisher's exact test.

### Bronchoscopy characteristics of asthmatic children with recurrent MLS

No significant differences were observed between the two groups in terms of macroscopic findings and BAL fluid inflammation types (Table 3). However, 10 children in the recurrent MLS group and 8 in the non-recurrent MLS group underwent follow-up bronchoscopy. A higher proportion of dilated bronchi was found in the recurrent MLS group than in the non-recurrent MLS group (60.0% vs. 12.5%, *P* = 0.066), even though the between-group difference was minimal. Children with severe asthma and recurrent MLS had similar macroscopic findings and BAL fluid inflammation types with severe asthma control (Table 4).

**Table 3:** Bronchoscopy findings of asthmatic children with RML and/or lingula atelectasis stratified by recurrent MLS.

Characteristics	Recurrent MLS	Non-recurrent MLS	*P*-value
**Macroscopic findings**	*n* = 15	*n* = 17	
Normal	1 (6.7%)	2 (11.8%)	1.000*
Mucus secretion	7 (46.7%)	7 (41.2%)	0.755
Fluid mucus secretion	7 (46.7%)	5 (29.4%)	0.314
Thick mucus secretion	0 (0.0%)	2 (11.8%)	0.486*
Mucus plug	2 (13.3%)	2 (11.8%)	1.000*
Mucosal inflammation	11 (73.3%)	12 (70.6%)	1.000*
Bronchial stenosis	3 (20.0%)	3 (17.6%)	1.000*
Bronchial obliteration	2 (13.0%)	0 0.0%)	0.212*
**BAL fluid inflammation type**	*n* = 15	*n* = 14	
Lymphocytic	4 (26.7%)	4 (28.6%)	1.000*
Neutrophilic	11 (73.3%)	11 (78.6%)	0.539*
Eosinophilic	4 (26.7%)	2 (14.3%)	0.651*

Data are presented as *n* (%). RML, right middle lobe; MLS, middle lobe syndrome; BAL, bronchoalveolar lavage.

* Comparisons were made by Fisher's exact test.

**Table 4:** Bronchoscopy findings of children with severe asthma stratified by recurrent MLS.

Characteristics	Severe asthma with recurrent MLS	Severe asthma control	*P*-value
**Macroscopic findings**	*n* = 11	*n* = 10	
Normal	0 (0.0%)	1 (10.0%)	0.476*
Mucus secretion	6 (54.5%)	3 (30.0%)	0.387*
Fluid mucus secretion	6 (54.5%)	3 (30.0%)	0.387*
Thick mucus secretion	0 (0.0%)	0 (0.0%)	
Mucus plug	1 (9.1%)	3 (30.0%)	0.311*
Mucosal inflammation	10 (90.9%)	9 (90.0%)	1.000*
Bronchial stenosis	3 (20.0%)	3 (17.6%)	1.000*
Bronchial obliteration	3 (27.3%)	1 (10.0%)	0.586*
**BAL fluid inflammation type**	*n* = 11	*n* = 9	
Lymphocytic	4 (36.4%)	2 (22.2%)	0.642*
Neutrophilic	8 (72.7%)	5 (55.6%)	0.642*
Eosinophilic	3 (27.3%)	4 (44.4%)	0.642*

Data are presented as *n* (%). MLS, middle lobe syndrome; BAL, bronchoalveolar lavage.

* Comparisons were made by Fisher's exact test.

### Lung function analysis of recurrent MLS with asthma

**Figure 1:**
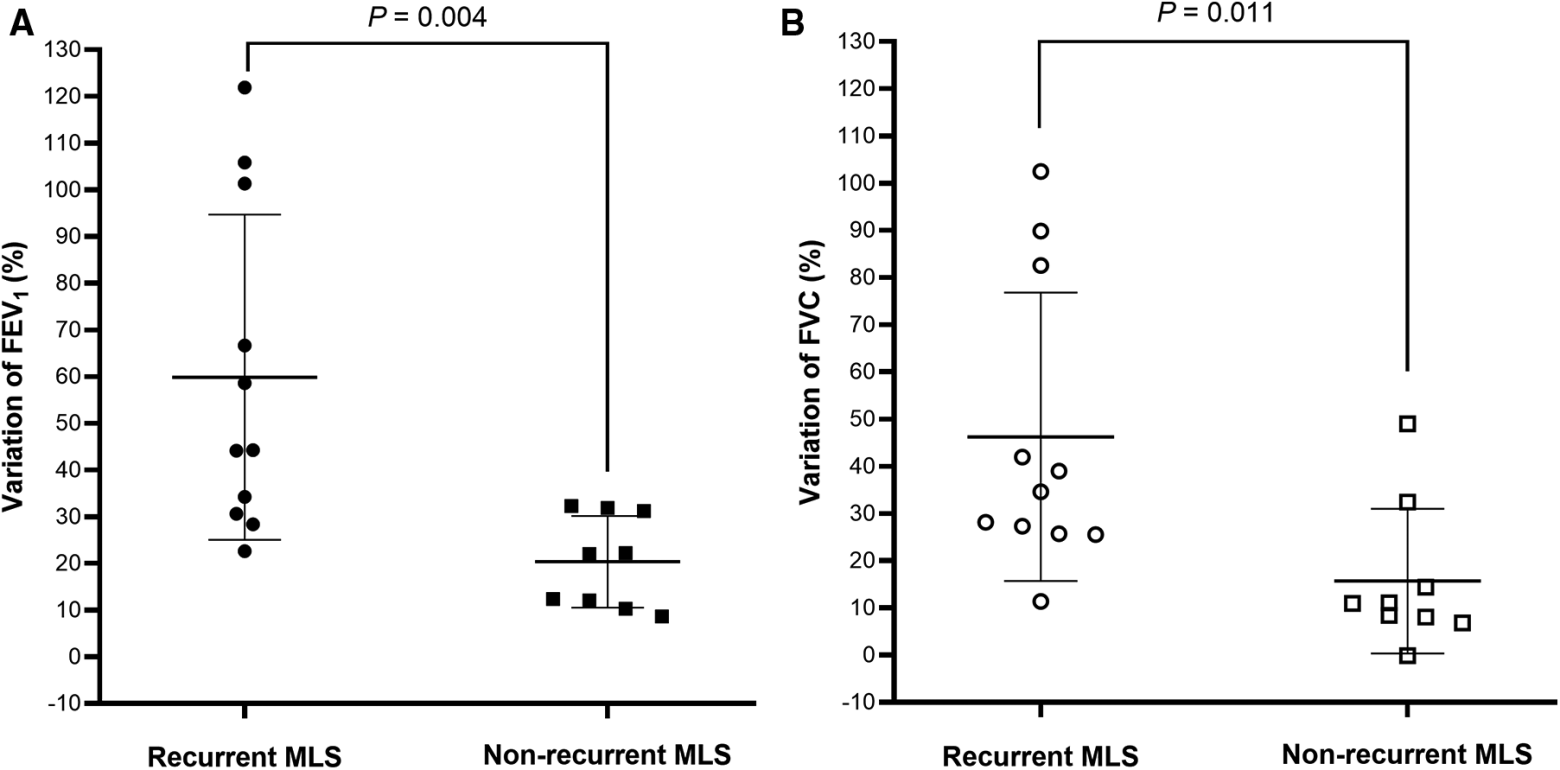
FEV_1_ (**A**) and FVC (**B**) variations between acute and stable stages were plotted for children with asthma with recurrent MLS vs. those with non-recurrent MLS. Bar charts represented mean ± standard deviation. FEV_1_: forced expiratory volume in 1 s; FVC: forced vital capacity; MLS: middle lobe syndrome.

Spirometry data, during the clinically stable stage, were available for 12 children with recurrent MLS and 10 non-recurrent MLS (Table 5). Compared to subjects with non-recurrent MLS, subjects with recurrent MLS had significantly higher % predicted and *z*-scores for pre-BD FEV_1_, FVC, FEF_25–75_, and FEF_75_, and a greater proportion of high FEV_1_. Interestingly, the variations in FEV_1_ (Figure 1A) and FVC (Figure 1B) between the acute and stable stages were significantly higher in the recurrent MLS group than in the non-recurrent MLS group. Children with recurrent MLS tended to have higher predicted values for post-BD FEV_1_ (*P* = 0.057), FVC (*P* = 0.080), and FEF_25–75_ (*P* = 0.081) than those with non-recurrent MLS.

**Table 5:** Spirometry of asthmatic children with RML and/or lingula atelectasis stratified by recurrent MLS.

Parameters	Recurrent MLS	Non-recurrent MLS	*P*-value
**Pre-BD**	*n* = 12	*n* = 10	* *
**FEV_1_**			
% predicted	105.5 ± 14.9	85.8 ± 11.5	0.005
≥100% predicted	8 (66.7%)	1 (10.0%)	0.011*
*z*-score	0.45 ± 1.27	−1.21 ± 1.00	0.006
Variation (%)^†^	59.9 ± 34.8 (*n* = 11)	20.4 ± 9.8 (*n* = 9)	0.004
**FVC**			
% predicted	109.2 ± 13.6	94.0 ± 8.9	0.013
≥100% predicted	9 (75.0%)	2 (20.0%)	0.010
*z*-score	0.89 ± 1.41	−0.64 ± 1.00	0.016
Variation (%)^†^	46.2 ± 30.6 (*n* = 11)	15.7 ± 15.3 (*n* = 9)	0.011
**FEV_1_/FVC**			
Ratio	87.6 ± 4.7	83.7 ± 10.9	0.371
% predicted	96.5 ± 4.7	91.9 ± 10.7	0.283
*z*-score	−0.59 ± 0.76	−1.12 ± 1.63	0.414
**FEF_25–75_**			
% predicted	96.8 ± 23.5	67.4 ± 24.5	0.015
*z*-score	−0.19 ± 1.06	−1.64 ± 1.35	0.015
**FEF_75_**			
% predicted	95.5 ± 20.3	64.2 ± 31.2	0.014
*z*-score	−0.21 ± 0.77	−1.66 ± 1.53	0.034
**Post-BD**	*n* = 8	*n* = 8	
FEV_1_% predicted	107.7 ± 16.5	91.9 ± 11.1	0.057
FVC % predicted	110.9 ± 18.0	95.8 ± 10.5	0.080
FEF_25–75_% predicted	114.8 ± 36.3	82.7 ± 25.8	0.081

Data are presented as *n* (%) or mean ± standard deviation. RML, right middle lobe; MLS, middle lobe syndrome; FEV_1_, forced expiratory volume in 1 s; BD, bronchodilator; FVC, forced vital capacity; FEF_25–75_, mean forced expiratory flow between 25% and 75% of FVC; FEF_75_, instantaneous forced expiratory flow at 75% of FVC.

* Comparisons were made by Fisher's exact test.

^†^ Variations were calculated as: (stable−acute)/mean (stable + acute) × 100.

**Figure 2:**
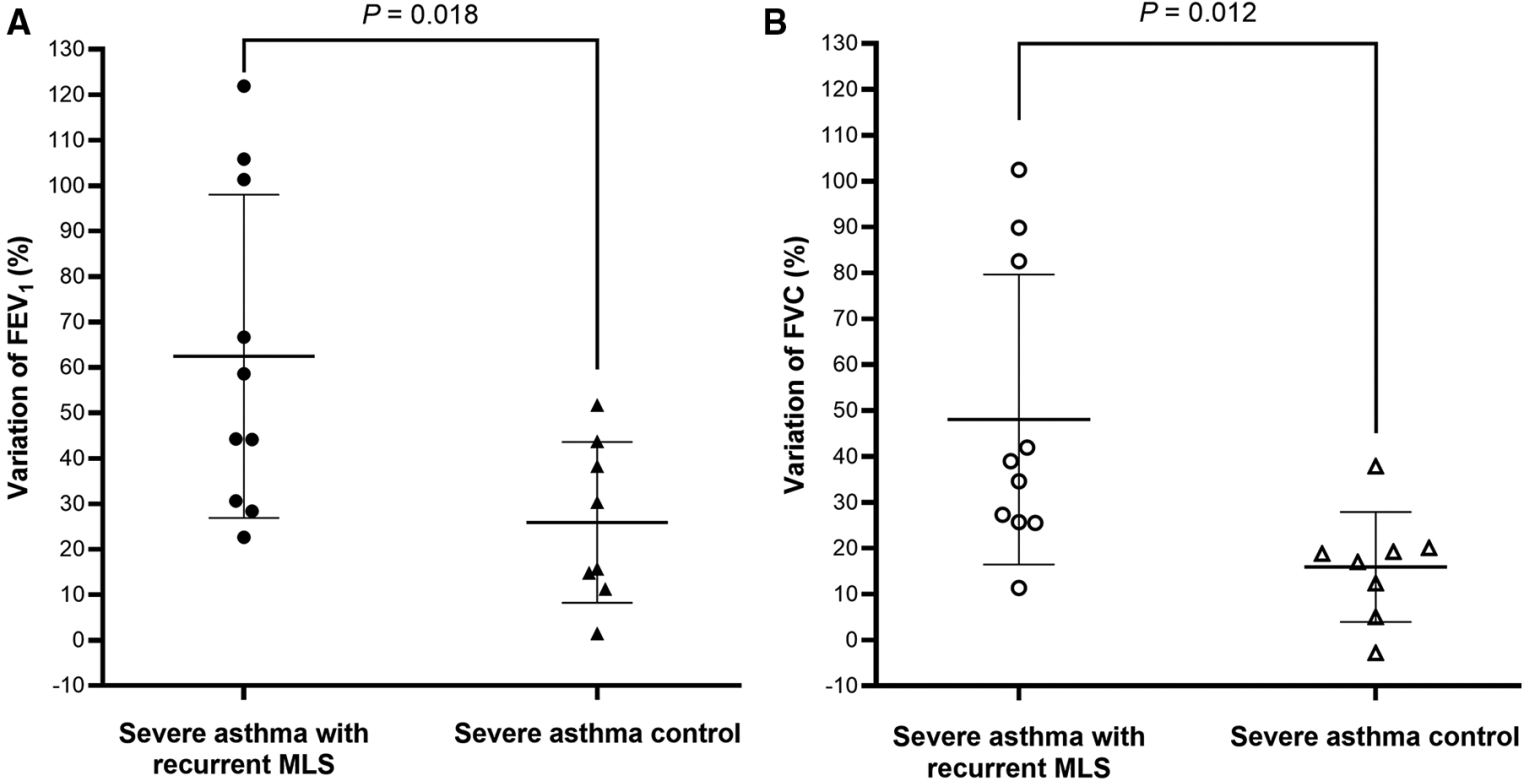
FEV_1_ (A) and FVC (B) variations between acute and stable stages are plotted for children with severe asthma with recurrent MLS vs. severe asthma controls. Bar charts represented mean ± standard deviation. FEV_1_: forced expiratory volume in 1 s; FVC: forced vital capacity; MLS: middle lobe syndrome.

Spirometry data, during the clinically stable stage, was available for 10 subjects with severe asthma and recurrent MLS, and 8 severe asthma controls (Table 6). Compared to severe asthma controls, subjects with recurrent MLS had significantly higher % predicted and *z*-scores for pre-BD FEV_1_, FVC, FEF_25–75_ and FEF_75_, a greater proportion of high FEV_1_, and higher variations of FEV_1_ (Figure 2A) and FVC (Figure 2B). Only the difference in the FVC *z*-score was minimally significant (*P* = 0.053).

**Table 6:** Spirometry of children with severe asthma stratified by recurrent MLS.

Parameters	Severe asthma with recurrent MLS	Severe asthma control	*P*-value
**Pre-BD**	*n* = 10	*n* = 8	
**FEV_1_**			
% predicted	108.3 ± 14.7	86.1 ± 12.9	0.004
≥100% predicted	8 (80.0%)	0 (0.0%)	0.001*
*z*-score	0.68 ± 1.26	−1.30 ± 1.23	0.004
Variation (%)^†^	62.5 ± 35.6	25.9 ± 17.7	0.018
**FVC**			
% predicted	111.7 ± 13.2	98.8 ± 9.5	0.033
≥ 100% predicted	8 (80.0%)	4 (50.0%)	0.321*
*z*-score	1.14 ± 1.40	−0.19 ± 1.27	0.053
Variation (%)^†^	48.0 ± 31.6	15.9 ± 12.0	0.012
**FEV_1_/FVC**			
Ratio	87.9 ± 5.0	78.1 ± 11.1	0.045
% predicted	96.6 ± 5.1	88.2 ± 10.8	0.070
*z*-score	−0.56 ± 0.83	−1.76 ± 1.57	0.077
**FEF_25–75_**			
% predicted	99.8 ± 24.8	67.4 ± 25.6	0.015
*z*-score	−0.05 ± 1.12	−1.86 ± 1.64	0.014
**FEF_75_**			
% predicted	99.4 ± 20.0	64.1 ± 24.8	0.004
*z*-score	−0.07 ± 0.76	−1.67 ± 1.37	0.014
**Post-BD**	*n* = 7	*n* = 7	
FEV_1_% predicted	110.4 ± 16.4	95.1 ± 9.2	0.059
FVC % predicted	115.3 ± 15.1	103.7 ± 13.1	0.167
FEF_25–75_% predicted	109.8 ± 37.1	84.0 ± 18.0	0.164

Data are presented as *n* (%) or mean ± standard deviation. MLS, middle lobe syndrome; FEV_1_, forced expiratory volume in 1 s; BD, bronchodilator; FVC, forced vital capacity; FEF_25–75_, mean forced expiratory flow between 25% and 75% of FVC; FEF_75_, instantaneous forced expiratory flow at 75% of FVC.

* Comparisons were made by Fisher's exact test.

^†^ Variations were calculated as: (stable−acute)/mean (stable + acute) × 100.

## Discussion

This retrospective analysis describes the characteristics of recurrent MLS caused by asthma in children. The most interesting finding was that, compared to asthmatic children with non-recurrent MLS, children with recurrent MLS had higher FEV_1_, FVC, FEF_25–75_ and FEF_75_, and a higher proportion of FEV_1_ and FVC ≥ 100% predicted during clinically stable stage, but more frequent severe asthma and severe exacerbations. It was also found that subjects with recurrent MLS had higher variations in FEV_1_ and FVC between acute asthma exacerbation and stable stages. During follow-up, more frequently dilated bronchi was found by bronchoscopy in patients with recurrent MLS. The finding of this phenotype of asthma with recurrent MLS is of significant clinical relevance, as these children are at greater risk of severe asthma and severe exacerbations, even with FEV_1_ and FVC_ _≥ 100% predicted when clinically stable.

Asthma with recurrent MLS might be a clinically significant phenotype, as these children are at a greater risk of severe asthma and exacerbations. MLS was not common in asthma, and it was reported that the incidence of MLS in children hospitalized with acute asthma was 5%–10% (21, 22). The present study showed that asthmatic children with recurrent MLS occurred more frequently in girls and in families with atopic history, and demonstrated higher levels of total IgE, and a greater proportion of severe asthma and exacerbations. The incidence of severe asthma in children with RML or lingula atelectasis was 34.3% (12/35), which was slightly higher than the 23.8% (5/21) in children with MLS, as reported by Springer et al. (8). A previous study found that children with severe asthma exhibited more frequent parental history of asthma, intubation, and higher serum IgE levels than those with mild to moderate asthma (23). However, on further comparing patients with severe asthma and MLS and severe asthma control, these differences disappeared. Therefore, the clinical characteristics of recurrent MLS with asthma may be due to a higher proportion of patients with severe asthma.

Furthermore, recurrent MLS and severe asthma still tended to occur more commonly in girls (6, 24). Girls are more susceptible to recurrent MLS because of their relatively smaller intraluminal diameters than boys (25). In addition, a slightly shorter duration of asthma treatment was observed in children with recurrent MLS and severe asthma, indicating the importance of early and regular use of asthma controller medications (2). Another possible reason is that the diagnosis of severe asthma was assessed retrospectively and later than the diagnosis of recurrent MLS. Further research is required to validate the role of the early use of asthma controller medications in the prevention of recurrent MLS and the predicted value of recurrent MLS for severe asthma.

Infections have close relationships with both asthma and atelectasis. In the present study, asthmatic children with recurrent MLS had higher incidence of infections than those with transient atelectasis. Bacterial infections have been suggested as a factor associated with persistent MLS in asthmatic children (8), but bacteria were only detected in three children with recurrent MLS in this study. The most common cause of infections was MP, which is one of the main causes of pneumonia in China (26). MP infections can cause serious airway mucosal damage, ciliary clearance dysfunction, epithelial cell shedding, and mucus plug, eventually leading to blockage of the bronchial lumen (27). However, the incidence of mucus plug was similar between severe asthma children with or without recurrent MLS. In addition, MP pneumonia is more likely to cause lower lobe consolidation or atelectasis (28, 29). Huang et al. reported 43 children with plastic bronchitis, pulmonary consolidation, and atelectasis, of which nearly half were caused by MP infections and 74.4% involved both lower lung lobes (29). Fever is common symptom of MP pneumonia especially the severe one with mucus plug (28, 29), but only occurred in 33.3% and 50.0% of children with recurrent MLS and non-recurrent MLS, respectively. And no differences were found in the incidence of fever between severe asthma children with or without recurrent MLS, suggesting that MP infection might not be the cause of recurrent MLS. Viral infection can also cause mucus plug and atelectasis, especially influenza virus (30, 31). In the present study, two cases with non-recurrent MLS had viral infections with influenza virus in only one case. Therefore, infection may be the trigger of acute asthma exacerbation, but not the reason of atelectasis or MLS.Interestingly, asthmatic children with recurrent MLS exhibited a distinct lung function phenotype with normal or even higher FEV_1_, FVC, FEF_25–75_, and FEF_75_ than those with non-recurrent MLS. Eight (66.7%) and nine (75.0%) asthmatic children with recurrent MLS had FEV_1_ and FVC ≥ 100% predicted respectively. However, this “normal” lung function did not indicate good outcomes as it neither controlled asthma nor severe exacerbations, even requiring PICU admission. A longitudinal study demonstrated that low lung function did not predict the persistence of severe asthma, whereas children with severe asthma and normal lung function did not experience improvement (32). Higher variations in FEV_1_ and FVC between acute and stable stages were also found in asthmatic children with recurrent MLS, which indicated airway instability. Consistent with our results, Sorkness et al. found that asthmatic children with a positive bronchodilator test assessed by FVC had more exacerbations and higher asthma severity scores (33). Comberiati et al. reported a special phenotype of severe asthma in children with FEV_1_ > 100% predicted during the clinically stable stage, who can experience life-threatening exacerbations (9). In addition, we observed that six asthmatic children with recurrent MLS had dilated bronchi during follow-up FOB. Therefore, the high lung function during the stable stage, catastrophic reductions during exacerbations, and dilated bronchi in follow-up FOB may be due to the loss of lung elasticity, uncoupling, and instability of airways.

This study had significant limitations related to its descriptive nature and retrospective design. Although the data were meticulously recorded, they were collected from electronic medical records, some of which were missing. Due to the retrospective analysis, half of the lung function data were missing in non-recurrent MLS group, which might have introduced a selection bias; however, the mean FEV_1_ (85.80% predicted) in non-recurrent MLS group was similar to the FEV_1_ (83.5% predicted) reported by Soyer et al. in asthmatic children with RML atelectasis (2). The same limitations accounted for difficulty in interpreting the presence of a high proportion of severe asthma but “normal” lung function in the recurrent MLS group; thus, we can only speculate that the patient had airway instability. Further prospective evaluations of this special clinical phenotype, including oscillometry and plethysmography, may help determine the lung mechanical properties in these children. Another limitation is the relatively small sample size, which made further multivariate logistic regression analysis impossible. The prevalence of MLS in asthmatic children has been low and has declined due to preventive anti-inflammatory therapies in recent years (6).

In conclusion, we showed that childhood asthma with recurrent MLS is a special phenotype with significant clinical relevance, with more frequent severe asthma and severe exacerbation but high lung function and variations. Further prospective studies are warranted to determine the prognosis of children with recurrent MLS or RML atelectasis.

## Data Availability

The raw data supporting the conclusions of this article will be made available by the authors, without undue reservation.
